# Clinical data analysis reveals the role of OGR1 (GPR68) in head and neck squamous cancer

**DOI:** 10.1002/ame2.12105

**Published:** 2020-03-18

**Authors:** Wenlong Zhang, Yong Han, Weisha Li, Lin Cao, Libo Yan, Chuan Qin, Ran Gao

**Affiliations:** ^1^ Key Laboratory of Human Disease Comparative Medicine (National Health and Family Planning Commission) The Institute of Laboratory Animal Science Chinese Academy of Medical Sciences & Peking Union Medical College Beijing P.R. China; ^2^ Beijing Engineering Research Center for Experimental Animal Models of Human Critical Diseases Beijing P.R. China; ^3^ Department of Pathology Zhejiang Provincial People’s Hospital Hangzhou Zhejiang P.R. China; ^4^ People’s Hospital of Hangzhou Medical College Hangzhou Zhejiang P.R. China; ^5^ Key Laboratory of Tumor Molecular Diagnosis and Individualized Medicine of Zhejiang Province Hangzhou Zhejiang P.R. China

**Keywords:** head and neck squamous cancer (HNSC), OGR1, TCGA, UALCAN

## Abstract

**Background:**

Head and neck squamous cancer (HNSC) frequently occurs in the clinic. Revealing the role of the genes that correlate with cancer cell outgrowth will contribute to potential treatment target identification and tumor inhibition.

**Methods:**

The gene expression profiles and gene ontology of the proton‐sensing G‐protein‐coupled receptor OGR1 were analyzed using the TCGA (The Cancer Genome Atlas) database. The effects of sex, age, race, and degree of malignancy on HNSC were investigated, and the survival times of HNSC patients with high or low/medium expression levels of OGR1 were compared. Methylation of the OGR1 promoter CpG sites was also investigated and OGR1‐related genes were analyzed using gene set enrichment analysis.

**Results:**

OGR1 is overexpressed in HNSC patients. However, compared with the low/median expression group, the high OGR1 expression group did not have different survival rates. The OGR1 expression level differed across sex, age, race, and degree of malignancy, while the methylation of the OGR1 promoter CpG sites was maintained at a similar level. Gene set enrichment analysis revealed that OGR1 was positively correlated with head and neck cancer, cisplatin resistance, hypoxia, angiogenesis, cell migration, and TGF‐β.

**Conclusion:**

The expression of OGR1 correlated with HNSC progression and survival and thus can serve as a potential treatment target and prognostic marker.

## 1 INTRODUCTION

HNSC is a common and aggressive malignancy with a high morbidity and mortality profile.^1^ Annually, approximately 600 000 people are diagnosed with head and neck squamous cancer worldwide. Thus far, biomarkers for HNSC are scarce; therefore, a better understanding of the dynamics between potential targets and HNSC is urgently needed.

OGR1 is a proton‐sensing G‐protein‐coupled receptor (GPCR) that plays an important role in extracellular acidity and cell stability, including roles in neurotransmission, sensory perception, cell metabolism, and differentiation.^2^

Expression of OGR1 is observed in many different human tissues, including brain, liver, heart, and spleen,^3^ and in cells such as thyroid cells, osteoblasts, osteocytes, chondrocytes, hepatocytes, aortic smooth muscle cells, airway smooth muscle (ASM) cells, T cells, and neutrophils.^4^, ^5^, ^6^, ^7^, ^8^, ^9^, ^10^, ^11^

In addition to its physiological function in normal tissues, numerous studies implicate OGR1 in important roles in the progression of different cancers. RNA‐seq data have shown that 12 subtypes of tumors have an approximately 2‐fold increase in OGR1 expression.^12^, ^13^ In addition to cancer cells, OGR1 may also be expressed in a variety of stromal cells of the TME (tumor microenvironment), including T cells, macrophages, endothelia cells, and cancer‐associated fibroblasts. The present data indicate that OGR1 represents a double‐edged sword; for instance, in prostate cancer, OGR1 has been shown to act as a metastasis‐suppressing gene,^14^ and high expression of OGR1 effectively increased MCF7 breast cancer cell apoptosis and inhibited MCF7 cell growth and proliferation,^15^ while other scientists have revealed that OGR1 can promote prostate cancer outgrowth.^16^ More studies are needed to illuminate the role of OGR1 in immune cells and cancer cells.

Using the TCGA database, the prognostic value of OGR1 expression in HNSC was investigated. The results indicate that OGR1 is overexpressed in HNSC patients and that its expression level is correlated with patient age, sex, race, and degree of malignancy. Thus, OGR1 may act as a potential therapeutic and prognostic target for HNSC.

## 2 METHODS

### 2.1 Ethics statement

All the data were retrieved from the online databases; thus, all written informed consent had already been obtained.

### 2.2 UALCAN

UALCAN (available online at http://ualcan.path.uab.edu/index.html) is an interactive web resource based on 3 RNA‐seq databases and the clinical data of 31 cancer types from the TCGA database. It can be used to analyze the relative transcriptional expression of potential genes of interest in tumor and normal samples and the association of transcriptional expression with related clinicopathologic parameters.^17^ Collection of tumor and control samples, and investigation of gene expression and methylation were as described in a previous publication.^18^ Briefly, each frozen tumor specimen had a companion normal sample which consisted of blood components, adjacent normal tissue more than 2 cm from the tumor, or previously extracted germline DNA from blood or normal tissue. RNA was extracted, collected into Illumina TruSeq mRNA libraries, sequenced by Illumina HiSeq2000 with a target of 60 million read‐pairs per tumor resulting in paired 48nt reads, and subjected to quality control, as previously described.^19^ RNA reads were aligned to the hg19 genome assembly using Mapsplice.^20^ Gene expression was quantified for the transcript models corresponding to the TCGA GAF2.1 using RSEM^21^ and normalized within‐sample to a fixed upper quartile of total reads. For further details on this processing, refer to the Description file at the DCC data portal under the V2_ MapSpliceRSEM workflow.^22^ For gene level analyses, expression values of zero were set to the overall minimum value, and all data were log2 transformed.^18^ Investigation of gene methylation was also conducted as described in a previous publication.^18^

Tumors were hierarchically clustered in R based on Euclidean distance using Ward's method. Fisher's exact *P* values were calculated for frequency comparisons of significantly reoccurring alterations by HPV status and site. For comparison of amplifications only high‐level events were considered (thresholded values = 2); for deletions all events were used.^18^

In this study, UALCAN was used to analyze the mRNA expression of the OGR1 gene in head and neck squamous cancer and its association with clinicopathologic parameters. The process of retrieval was performed according to the software tutorial. GC data from The Cancer Genome Atlas (TCGA) were downloaded to determine the OGR1‐related genes that were used for gene set enrichment analysis (GSEA) and BiNGO analysis. The proteins that physically interacted with OGR1 and drugs that could potentially upregulate OGR1 were obtained from CTDbase.

### 2.3 Statistical analysis

All data are presented as the mean ± SD, and significant differences between the two groups were determined using the Mann‐Whitney U test. *P* < .05 was defined as statistically significant.

## 3 RESULTS

### 3.1 OGR1 is overexpressed in HNSC patients

**FIGURE 1:**
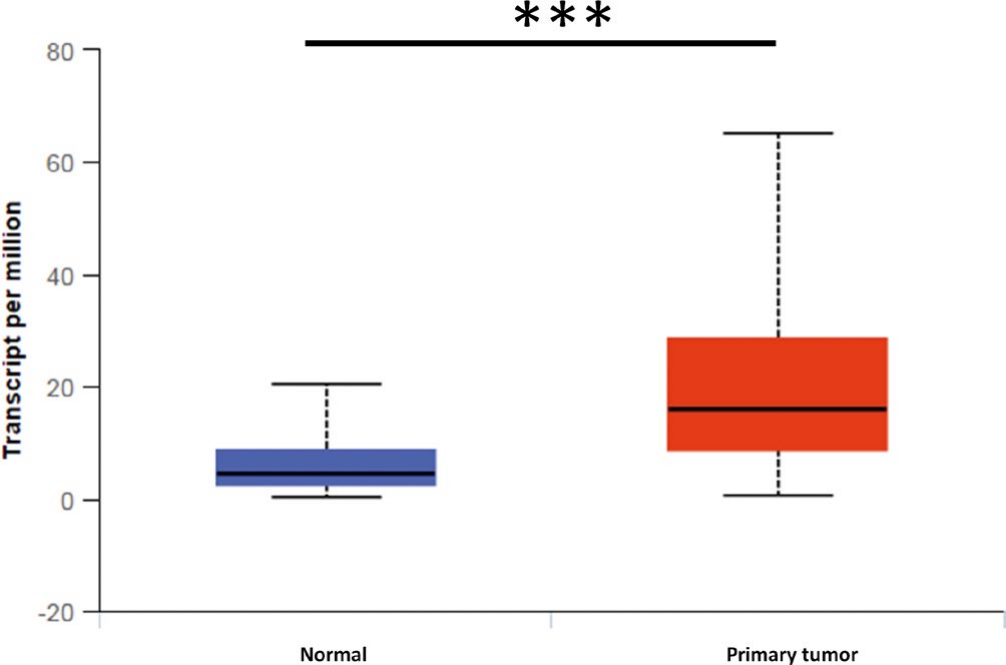
OGR1 is overexpressed in primary HNSC. Compared with the expression in normal tissues (n = 44), the expression of OGR1 in primary HNSC tissues (n = 520) is significantly higher (***P < .001)

First, the gene expression of OGR1 in HNSC patients was analyzed by comparing normal tissue and primary tumor samples. As shown in Figure 1, the expression of OGR1 was significantly higher in primary HNSC tumors than in control tissues (*P* = 1.63 × 10^−12^ < .001). This result illustrates that OGR1 is overexpressed in HNSC patients and is correlated with HNSC progression.

### 3.2 OGR1 expression differs with patient sex, age, race, and degree of malignancy

**FIGURE 2:**
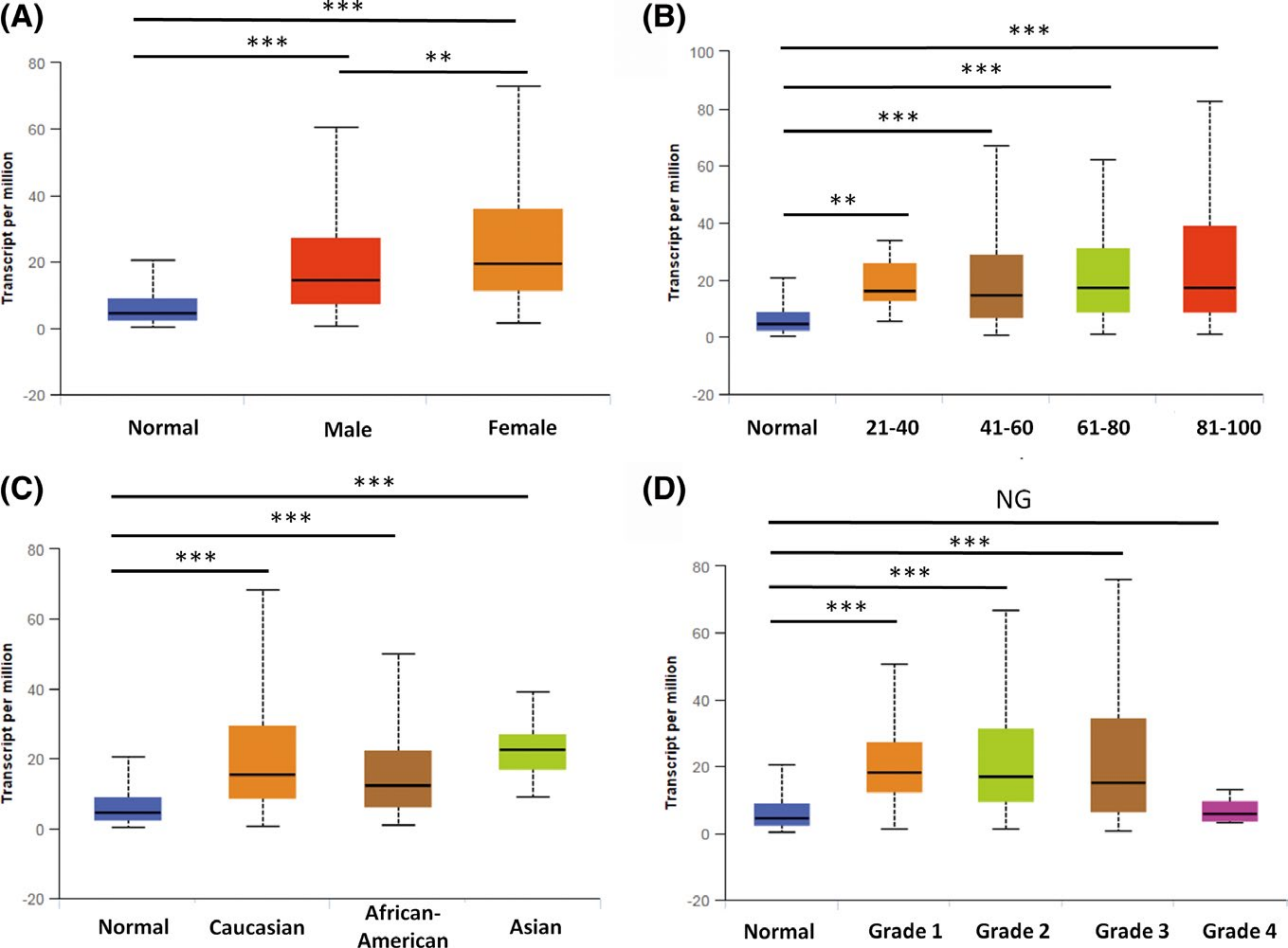
OGR1 expression differs in different groups of HNSC patients. A, There were statistical differences in OGR1 expression between male and female HNSC patients (normal n = 44, male n = 383, female n = 136). B, The expression of OGR1 in HNSC patients of different ages was higher than that in the controls (normal: n = 44, 21‐40: n = 20, 41‐60: n = 236, 61‐80: n = 237, 81‐100: n = 24). C, OGR1 expression was higher in Caucasian (n = 444), African‐American (n = 47), and Asian (n = 11) patients than in the controls (n = 44). D, OGR1 expression was higher in grade 1 (n = 62), grade 2 (n = 303), and grade 3 (n = 125) disease than in the controls (n = 44), while grade 4 (n = 7) tissues had similar levels of OGR1 expression to the controls. (**P < .01, ***P < .001)

OGR1 expression in patients of different sex, age, race, and degree of malignancy were analyzed (Figure 2). As shown in Figure 2A, the expression of OGR1 was significantly higher in male (*P* = 2.28 × 10^−12^ < .001) and female (*P* = 1.63 × 10^−12^ < .001) HNSC patients than in normal tissue. Compared with the expression in male patients, OGR1 expression was much higher in female patients (*P* = 8.44 × 10^−3^ < .01).

OGR1 expression also differed across patient age ranges (Figure 2B). Compared with the control groups, significantly higher expression of OGR1 was seen in HNSC patients across the following age ranges (years): 21‐40 (*P* = 2.34 × 10^−3^ < .001), 41‐60 (*P* = 2.71 × 10^−12^ < .001), 61‐80 (*P* = 1.58 × 10^−13^ < .001), and 81‐100 (*P* = 9.24 × 10^−4^ < .001). Patients aged 21‐40 had the highest expression of OGR1, while patients aged 41‐60 had the lowest expression, but there were no significant differences in expression between the different age groups of patients. This indicates that the expression of OGR1 differs with the age of the patients; however, the differences in expression are not significant.

The data also show that expression of OGR1 differs between races (Figure 2C): Caucasian (*P* = 1.63 × 10^−12^ < .001), African‐American (*P* = 5.40 × 10^−4^ < .001), and Asian (*P* = 2.01 × 10^−6^ < .001) HNSC patients presented significantly higher expression of OGR1 than normal samples. However, there was no significant difference between expression between these three races (Caucasian versus African‐American *P* = .429 > .05, Caucasian versus Asian *P* = .77 > .05, African‐American vs Asian *P* = .45 > .05).

OGR1 expression was also analyzed across different degrees of malignancy in HNSC patients (Figure 2D). The expression of OGR1 in grade 1‐3 disease was significantly higher (grade 1 vs control *P* = 1.15 × 10^−8^ < .001, grade 2 vs control *P* = 2.44 × 10^−15^ < .001, grade 3 versus control *P* = 8.00 × 10^−10^ < .001) than the expression in the controls. However, the expression of OGR1 in grade 4 patients was similar to that in the controls (*P* = .42 > .05). In these groups, the expression of OGR1 was highest in grade 1 disease, and the higher the tumor malignancy, the lower the OGR1 expression.

### 3.3 Similar survival times were found in the high and low OGR1 expression groups

**FIGURE 3:**
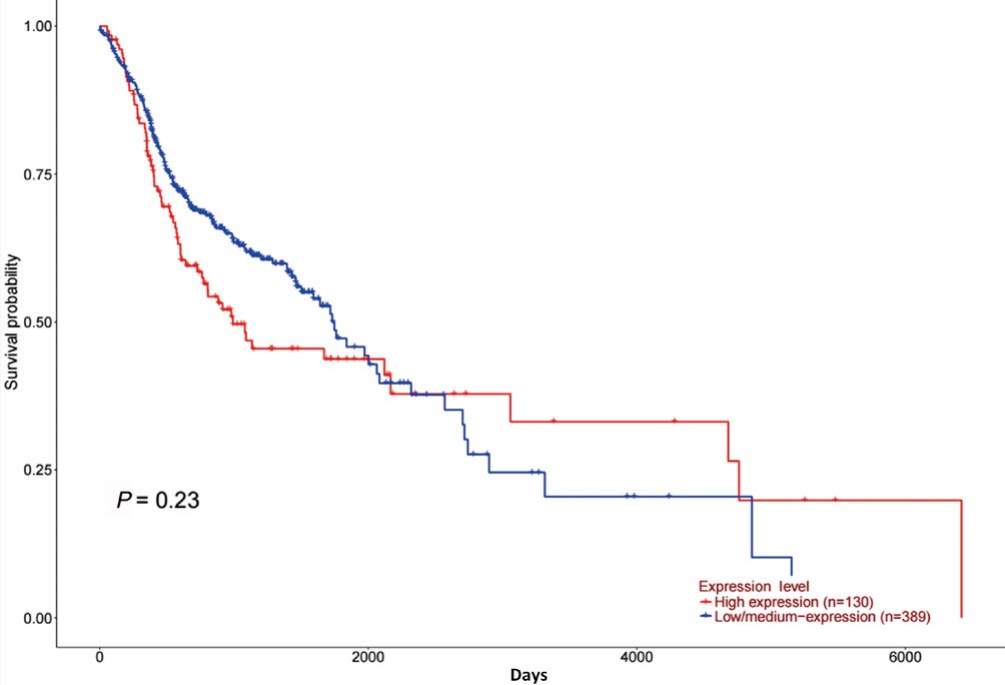
Comparison of the survival time between the high and low/medium OGR1 expression groups in HNSC patients. HNSC patients with high expression of OGR1 (n = 130) survived longer than low/medium OGR1 expression HNSC patients (n = 389)

To investigate the associations between the gene expression of OGR1 and overall survival, survival curves were analyzed for the low/medium OGR1 expression group and the high expression group (Figure 3). There was no significant difference between the two groups (*P* = .23 > .05). This indicates that HNSC patients with lower expression of OGR1 had similar overall survival to those patients with high OGR1 expression.

### 3.4 Promoter CpG site methylation was maintained at a stable level in groups of HNSC patients of different sex, age, race, and degree of malignancy

**FIGURE 4:**
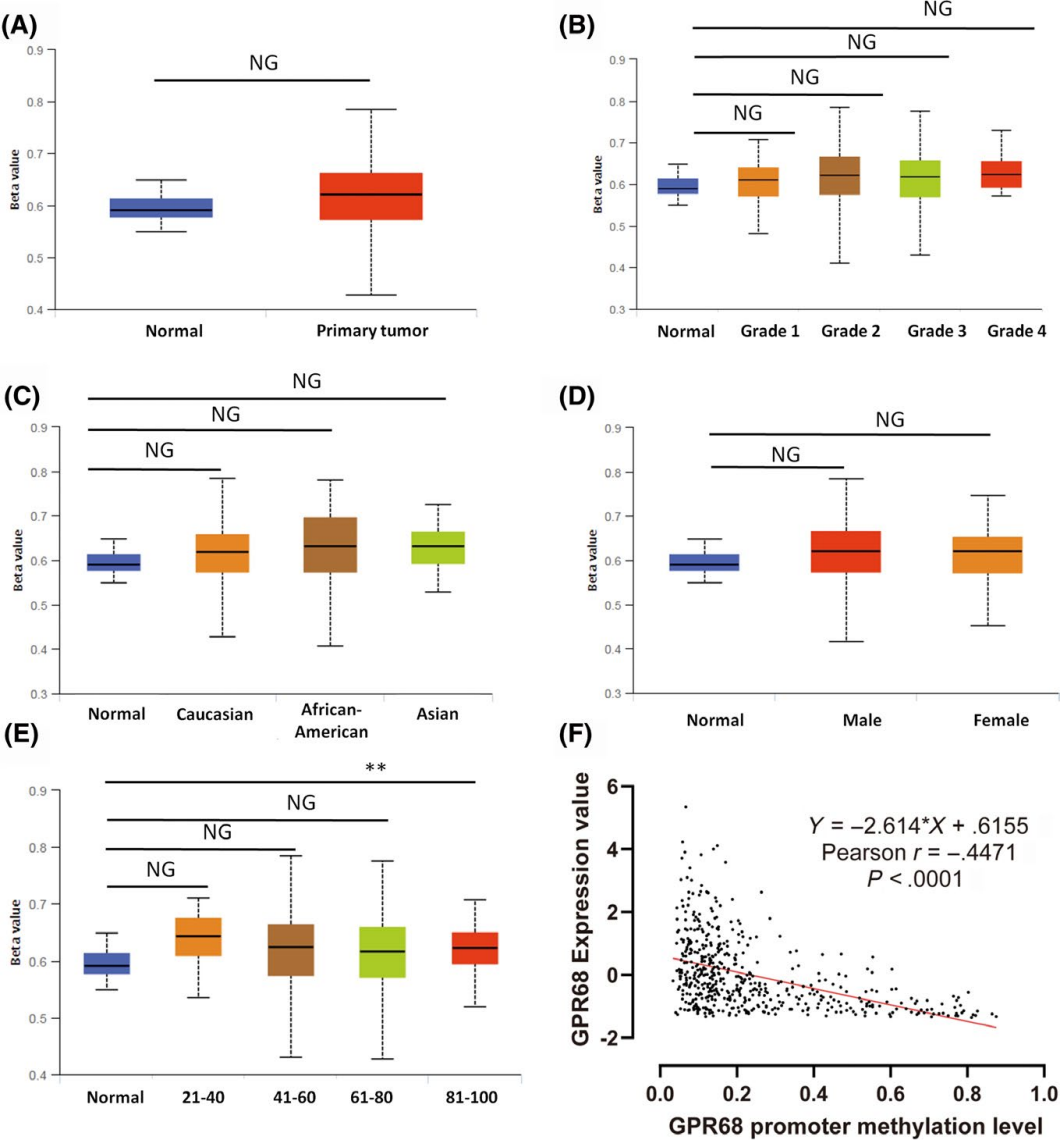
OGR1 promoter CpG site methylation remains at a stable level across different groups of HNSC patients. A–E, The OGR1 promoter CpG sites methylation was maintained at a relatively stable level across different degrees of malignancy, race, sex, and age (the n is the same as that in each group in Figure 2) (*P < .05). F, OGR1 expression was negatively correlated with promoter CpG site methylation

The promoter CpG site methylation of OGR1 was also investigated (Figure 4). Compared with the level in normal samples, the OGR1 promoter CpG site methylation level was similar in HNSC tumor tissues (Figure 4A, *P* = .33 > .05). Similar results were also obtained for other clinicopathological parameters: tumor grade (Figure 4B), race (Figure 4C), sex (Figure 4D), and age (Figure 4E). One exception was in elderly patients, in which the level of promoter CpG site methylation was significantly higher than that in normal samples (Figure 4E, *P* = .017 < .05, 81‐100 years old). Additionally, the level in the 81‐ to 100‐year‐old patients was significantly higher than that in the 41‐ to 60‐year‐old patients (Figure 4E, *P* = .036 < .05) and the 61‐ to 80‐year‐old patients (Figure 4E, *P* = .039 < .05). Figure 4F illustrates that the expression of OGR1 is negatively correlated with promoter CpG site methylation.

### 3.5 Gene expression and methylation of GPR68 was not significantly correlated with patient malignancy, age and overall survival

**FIGURE 5:**
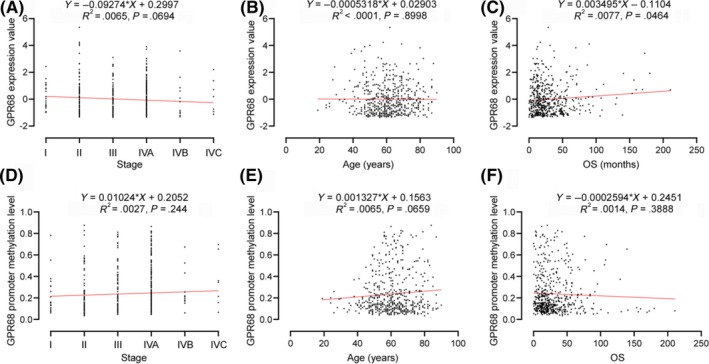
Regression analysis of correlation between GPR 68 gene expression, methylation and patients’ age, malignancy and survival. A‐C, Correlation between gene expression of GPR68 and tumor malignancy, age, and patients’ overall survival. D‐F, Correlation between methylation of GPR68 and tumor malignancy, age, and patients’ overall survival

Although OGR1 expression is significantly higher in HNSC patients, it is not significantly correlated with patient malignancy, age and overall survival (Figure 5A–C). Similarly, there was no significant correlation between OGR1 methylation and patient malignancy, age and overall survival (Figure 5D–F).

### 3.6 Functional enrichment analysis of OGR1‐associated genes

**FIGURE 6:**
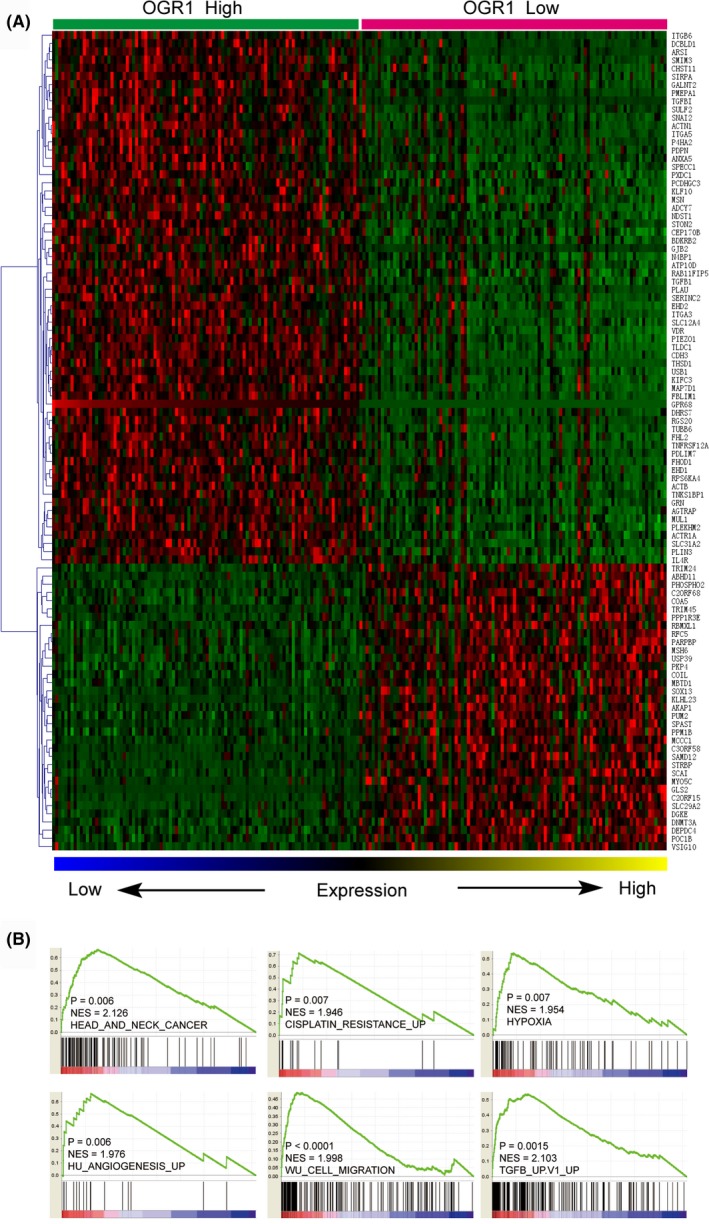
Molecular mechanisms underlie OGR1‐associated HNSC progression. A, The heat map shows 100 differentially expressed genes between the top and bottom 100 samples of HNSC ranked by OGR1 expression. B, OGR1 expression is positively correlated with HNSC, cisplatin resistance, hypoxia, angiogenesis, migration, and TGF‐β

To investigate the effects of OGR1 on other genes, the function of OGR1‐associated genes was analyzed. Each dataset was ranked from high to low, and differentially expressed genes were computed by comparing the top 100 samples with the bottom 100 samples; the 100 genes that had the most significant expression differences are shown in Figure 6A.

Figure 6B illustrates that OGR1 is positively correlated with HNSC, cisplatin resistance, hypoxia, angiogenesis, cell migration, and TGF‐β.

## 4 DISCUSSION

OGR1 is a pH‐sensing GPCR involved in maintaining pH homeostasis, functioning as a proton‐sensing receptor that stimulates inositol phosphate formation at pH < 6.8.^5^ Activation of OGR1 has been shown to produce a protumorigenic response that includes activation and secretion of proinflammatory proteins such as COX2, IL‐6, and IL‐8, which assist tumor progression.^23^, ^24^, ^25^ OGR1 expression is highly upregulated in numerous types of cancer, and previous studies provide evidence for OGR1 as a potentially novel therapeutic target.^26^ Although the role of OGR1 in certain cancers has been identified, its role in HNSC is still unclear.

We analyzed the association between the mRNA expression of OGR1 and different clinicopathologic parameters, including tumor grade, sex, race, and age. We also investigated the promoter CpG site methylation of OGR1 using UALCAN. The results indicate that OGR1 mRNA expression is significantly higher in HNSC patients than in normal samples; however, high OGR1 expression did not affect patient survival time.

DNA methylation is an epigenetic modification that has been extensively studied,^27^ and it is also associated with clinicopathological features and poor prognosis.^28^, ^29^ Our data illustrate that OGR1 DNA promoter CpG site methylation in HNSC is maintained at a relatively stable level, with no significant differences seen between HNSC and normal samples. This suggests that overexpression of OGR1 is not due to DNA methylation. Although OGR1 expression is significantly higher in HNSC patients, regression analysis shows that no significant differences between OGR1 gene expression and tumor malignancy, patients’ age and overall survival are apparent.

The reasons for the OGR1 expression differences remain unknown and need to be investigated. Previous studies also suggest that OGR1 expression in myeloid‐derived cells, particularly in DP (CD11b^+^Gr1^+^) cells, is important for tumor cell‐induced immunosuppression and tumor development. Moreover, OGR1 may promote macrophage phenotype transformation and T‐cell infiltration in prostate cancer. The mechanisms driving the effect of OGR1 on HNSC immunity require further investigation.

In summary, we analyzed the role of OGR1 in HNSC using the TCGA database, and our data indicate that OGR1 may serve as a target for developing new therapeutic approaches and early detection.

## 5 CONFLICT TO INTEREST

None.

## AUTHOR CONTRIBUTIONS

WZ, YH, WL, GZ, LC, XY, ML, HL, LW, CQ, and RG carried out the data analysis and figure preparation. RG and WZ prepared the manuscript. All authors read and approved the final manuscript.
